# Antiviral Therapy and Outcomes of Patients with Pneumonia Caused by Influenza A Pandemic (H1N1) Virus

**DOI:** 10.1371/journal.pone.0029652

**Published:** 2012-01-20

**Authors:** Shi-gui Yang, Bin Cao, Li-rong Liang, Xiao-li Li, Yong-hong Xiao, Zhi-xin Cao, Hong-yu Jia, Hong-jie Yu, Zhen Xu, Li Gu, Yi-da Yang, Yu Chen, Wei-bo Du, Xi-xin Yan, Zong-an Liang, Wei Zhang, Chang-le Zhang, Wei Chen, Cai-ping Guo, Xun-liang Jiang, Ming Yang, Guang-ming Deng, Kai-jiang Yu, Ke Hu, Qi Zou, Lan-juan Li, Chen Wang, for the National Influenza A Pandemic (H1N1) 2009 Clinical Investigation Group of China

**Affiliations:** 1 State Key Laboratory for Diagnosis and Treatment of Infectious Diseases, The First Affiliated Hospital, School of Medicine, Zhejiang University, Key Laboratory of Infectious Diseases, Hangzhou, Zhejiang Province, China; 2 Beijing Chao-Yang Hospital, Beijing Institute of Respiratory Medicine, Beijing Key Laboratory of Respiratory and Pulmonary Circulation, Capital Medical University, Beijing, China; 3 Disease Control and Emergency Response Office, Chinese Center for Disease Control and Prevention, Beijing, China; 4 Department of Respiratory Medicine, Second Hospital of Hebei Medical University, Shijiazhuang, China; 5 West China Hospital of Sichuan University, Chengdu, China; 6 The First Affiliated Hospital, Nanchang University. Nanchang, China; 7 Wenzhou No.2 People's Hospital, Wenzhou, China; 8 Shengjing Hospital of China Medical University, Shenyang, China; 9 Capital Medical University Beijing You'an Hospital, Beijing, China; 10 The Children's Hospital of Hunan Province, Changsha, China; 11 Beijing Children's Hospital, Beijing, China; 12 Shaoyang Central Hospital, Shaoyang, China; 13 The Second Affiliated Hospital of Harbin Medical University, Harbin, China; 14 Remin Hospital of Wuhan University, Wuhan, China; 15 Fujian Medical University Union Hospital, Fuzhou, China; Centro Nacional de Microbiología - Instituto de Salud Carlos III, Spain

## Abstract

**Background:**

There is limited data on the clinical outcome of patients with pandemic H1N1 (pH1N1) pneumonia who received oseltamivir treatment, especially when the treatment was administered more than 48 hours after symptom onset.

**Methods:**

During the pandemic in 2009, a cohort of pH1N1 influenza pneumonia was built in China, and their clinical information was collected systematically, and analyzed with Cox models.

**Results:**

920 adults and 541 children with pneumonia who didn't receive corticosteroids were analyzed. In-hospital mortality was higher in adults who did not receive antiviral therapy (18.2%) than those with who received oseltamivir ≤ 2days (2.9%), between 2–5 days (4.6%) and >5 days after illness onset (4.9%), p<0.01. A similar trend was observed in pediatric patients. Cox regression showed that at 60 days after symptoms onset, 11 patients (10.8%) who did not receive antivirals died versus 4 (1.8%), 18 (3.3%), and 23 (3.7%) patients whose oseltamivir treatment was started ≤ 2days, between 2–5days, and >5 days, respectively. For males patients, aged ≥ 14 years and baseline PaO_2_/FiO_2_<200, oseltamivir administration reduced the mortality risk by 92.1%, 88% and 83.5%, respectively. Higher doses of oseltamivir (>3.8 mg/kg/d) did not improve clinical outcome (mortality, higher dose 2.5% vs standard dose 2.8%, p>0.05).

**Conclusions:**

Antiviral therapy might reduce mortality of patients with pH1N1 pneumonia, even when initiated more than 48 hours after onset of illness. Greater protective effects might be in males, patients aged 14–60 years, and patients with PaO_2_/FiO_2_<200.

## Introduction

In early April 2009, human infections caused by influenza A pandemic H1N1 (pH1N1) 2009 virus were identified in the United States [1] and Mexico [2] and spread rapidly to other regions of the world, resulting in the first influenza pandemic since 1968 [3]. As of March 2010, almost all countries had reported laboratory-confirmed cases, and more than 17,700 deaths had been reported to the World Health Organization (WHO) [4]. pH1N1 virus infection causes disease requiring hospitalisation of previously fit individuals as well as those with underlying conditions [5]. In the United States, an estimated 59 million illnesses, 265,000 hospitalizations, and 12,000 deaths had been caused by the 2009 H1N1 virus as of mid-February 2010 [6]. In mainland China, there were more than 127,000 laboratory confirmed cases and 793 deaths as of February 28, 2010 [7].

Currently, no randomized controlled trial (RCT) of neuraminidase-inhibitor treatment of patients with influenza viral pneumonia has been conducted. Observational studies have suggested that oseltamivir therapy of adults hospitalized with seasonal influenza (22%–43% of these patients had viral pneumonia) may reduce mortality [8]–[10]. During this pandemic, although antiviral therapy was recommended [11], evidence was still limited about the correlation between oseltamivir treatment and clinical outcome, including hospitalization [12], admission to intensive care units (ICUs), and even death [13]–[15], especially for patients with pH1N1 pneumonia who were started on antiviral therapy >48 hours after illness onset [16].

During this pandemic, the National Influenza A pH1N1 2009 Clinical Investigation Group of China screened 3570 hospitalized patients with pH1N1 virus infection, and at last built a cohort of 3066 patients with pneumonia caused by 2009 pH1N1 virus. This large database gave us the opportunity to assess the effectiveness of oseltamivir treatment for pneumonia caused by 2009 pH1N1 virus. We also analyzed the optimal timing and dosing of oseltamivir in the treatment of 2009 pH1N1 pneumonia both in adults and in children.

## Methods

### Data sources

Participating centers were identified by the National Influenza A pH1N1 2009 Clinical Investigation Group of China. This is a national network for the diagnosis and treatment of pH1N1, and includes the Chinese Disease Control and Prevention (CDC) and community hospitals and teaching hospitals around China that are under the guidance by the Chinese Ministry of Health (MOH). Hospitalized patients were included in this study if they met the diagnostic criteria of having new radiographic abnormality indicating pneumonia with laboratory-confirmed case of pH1N1 virus between September 1 and December 31, 2009. Pneumonia was defined as an acute lower respiratory tract illness with two or more of the following symptoms or signs: cough, productive sputum, fever, chills, dyspnea, pleuritic chest pain, crackles, and bronchial breathing plus an opacity or infiltrate seen on a chest radiography that was interpreted as pneumonia by the treating physicians. Both adult and child inpatients were included. According to the pH1N1 2009 Clinical guideline (Third Edition, 2009) released by China MOH, a severe or critical case was defined as those who met at leaset one of the following criteria on admission: (1) respiratory failure; (2) septic shock; (3) multiple organs insufficiency; (4) other critical clinical conditions requiring intensive care. Hospitalized patients were excluded if they did not have pneumonia. Patients were also excluded if they had been treated as outpatients or in emergency rooms, had a duration of hospitalization <24 hours, or if there was an incomplete record of clinical outcome [17]. All patients who were treated with corticosteroids were also excluded in order to reduce any bias from corticosteroids treatment as far as possible.

### Antiviral therapeutic regimen

Based on local guidelines, antiviral therapy was considered for severe cases and high risk patients who had been infected with pH1N1 virus within 48 hours from onset of illness, no matter they had pneumonia or not. For patients who presented pH1N1 influenza-like symptoms as fever (> = 38°C), cough, and sore throat for more than 2 days, the managing physicians were allowed to make their own decisions regarding antiviral use. Oseltamivir, for patients older than 12 years, was prescribed according to the standard dosing regimen (75 mg twice daily orally, for 5 days). Dosage adjustment, if necessary, was made according to the patient's renal function (75 mg daily, if creatinine clearance <30 ml/min). For children (1–12 years of age), dosage adjustment was made according to body weight (BW), that is: 30 mg Bid for children with BW<15 kg, 45 mg Bid for BW 15–23 kg, 60 mg Bid for BW 23–40 kg, 75 mg Bid for BW>40 kg.

Oseltamivir treatment was defined as at least 1 day of drug therapy received by a patient. The time interval between symptom onset and the administration of the first dose of oseltamivir was calculated for each patient. The definition of standard dose was oseltamivir ≤ 3.8 mg/kg/d, and a higher dose oseltamivir >3.8 mg/kg/d for more than 3 days (**Figure S2**).

### Laboratory Confirmation

Pharyngeal or nasopharyngeal swabs were collected from all patients. We used the protocol set by the US Centers for Disease Control and Prevention, real-time RT-PCR for swine influenza A (H1N1) as recommended by the WHO [18].

### Data collection

Data collection and analysis were coordinated by the Chinese MOH. A standard data collection form was used at each site. Site investigators were primarily infectious disease physicians closely involved in taking care of such patients at their centers. They were asked to submit all cases that had been noted based on above inclusion and exclusion criteria. Clinical information was collected systematically from admission to discharge for each patient. A trained team of physicians and medical students reviewed the patient charts and recorded demographic, clinical, and laboratory information, chest X-ray, results of diagnostic testing for influenza, antiviral and corticosteroid treatment, non-invasive or invasive ventilation, clinical complications and outcome. The data was entered in duplicate into a computerized database. The data were analyzed anonymously. The research ethics board at Beijing Chao-Yang Hospital and First Affiliated Hospital, School of Medicine, Zhejiang University approved the study, and the consent statement was in written form.

### Statistical Analysis

The main outcome was all-cause mortality that occurred during a given hospital stay.

Means (standard deviations) or medians (interquartiles, IQR) were calculated as summaries of continuous variables. For categorical variables, percentages of patients in each category were calculated. We compared clinical characteristics and clinical outcomes by an ANOVA test, chi-square test, and Fisher's exact test, as appropriate. Cox model with time-varying treatment variable; categorical variable for time from symptom-onset; and an interaction between these two variables was performed to identify the protective effects of oseltamivir and to avoid survivor bias. In the model, survival time was treated as time variable, and death as status, antiviral therapy, its initial time and its interaction with initial time as covariates. A survival plot, separate lines for antiviral therapy and its initial time, was used to identify the protective effects of oseltamivir in the study. All analyses were carried out using SPSS for Windows (release 13.0).

## Results

### Clinical description of cohort

**Figure 1:**
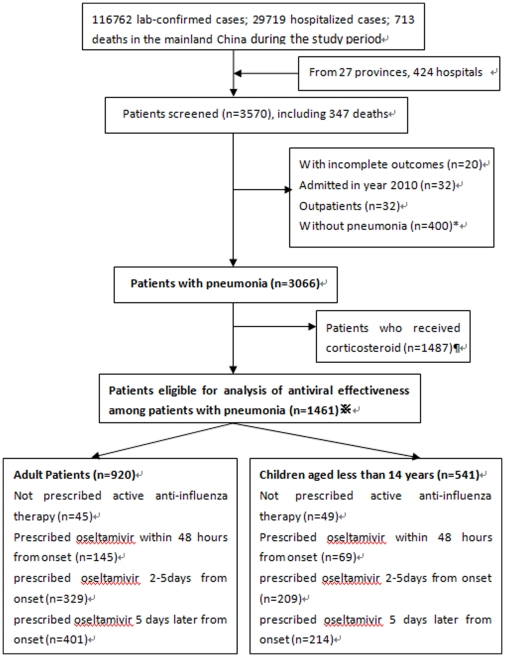
Flow chart of patients enrolled and included in the analysis of the impact of antiviral therapy on mortality. *Missing data for pneumonia (n = 20); ¶missing data for corticosteroid (n = 9); ※missing data for timing or whether oseltamivir was prescribed (n = 109).

Altogether, 3570 hospitalized cases were screened among them 3066 patients had pneumonia. The patient cohort was identified from 424 hospitals in 27 provinces in mainland China. This cohort represented approximately 11.7% of all patients hospitalized for pH1N1, and 48.7% (n = 347) of all deaths due to pH1N1 in mainland China during the study period (lab-confirmed cases 116762; hospitalized cases 29719; death cases 713). After exclusion patients who received corticosteroids, a total of 1461 patients (920 adults and 541 children) were included in the final analysis on the effects of antiviral therapy on mortality. **(** **Figure 1** **)**.

Among 1461 patients, the mean age was 23.3(IQR: 5.2–41.3) years and 55.2% (807) were male. 8.0% (49) had a BMI >30 kg/m2 The main coexisting diseases were cardiovascular diseases, respiratory diseases, and diabetes mellitus, accounting for 13.4% (207), 10.6% (164) and 5.9% (91), respectively. Pregnancy and postpartum accounted for 7.8% (119) and 2.9% (44). Antibiotics, oseltamivir, traditional Chinese medicine, antiviral plasma, or convalescent plasma were prescribed to97.6% (1426), 93.6% (1367), 51.5% (750), and 2.2% (32), respectively. 9.8% (143) were mechanically ventilated. The median length of hospitalization was 9 days and the in-hospital mortality was 3.9%, with the highest mortality among patients ≥60 yrs of age (**Table S1**).

### Clinical outcomes and antiviral therapy among patients with pneumonia

#### Description of basic conditions among the different groups of antivirals

Detailed clinical data was available for 920 adults and 541 children (less than 14 years of age). The median age of adults was 35.1 years (range 14–99 years). **(Table S2)** Those given oseltamivir >5 days were older than patients given oseltamivir 2–5 days after onset of illness (41.2 vs 36.7 years, p<0.05). More pregnant women were given antiviral therapy than non-pregnancy (p<0.05). There was no difference between the four treatment groups (divided based on when/if they received oseltamivir therapy) concerning BMI, smoking status, common features of illness (hemoptysis, dyspnea, CNS symptoms and leucopenia), complications from illness (ARDS, septic shock, acute renal failure, liver damage, and bacterial co-infection), APACHE II score, and SOFA score 24 hours after hospital admission. Except for oseltamivir, the frequency of administration of other treatments, including antibiotics, traditional Chinese Medicine, oxygen therapy, and convalescent plasma was similar between groups (p>0.05).

#### Comparison of clinical outcomes among the different groups of antivirals

The in-hospital mortality was higher in patients who did not receive antiviral therapy (18.2%, 8 died) than those who received oseltamivir ≤ 2days (2.9%, 4), between 2–5 days (4.6%, 15) and >5 days (4.9%, 18), p<0.01. After excluding patients who died within 96 hours of illness onset, the in-hospital mortality among the four groups was 16.3% (7), 2.9% (4), 4.6% (15) and 4.9% (18), respectively (P<0.01).

However, taken comparison among the three treatment groups into consideration, the in-hospital mortality had not significant difference. But more patients who received oseltamivir >5 days needed intubation and mechanical ventilation compared to those who received oseltamivir ≤ 2days (P<0.01) and 2–5 days after illness onset (P<0.01). There was no difference in ICU admission rates between the four groups.

The median age of children was 4.0 years (range, 27 days–14 years). **(Table S3)** Univariate analysis indicated that the trend of in-hospital mortality between the four treatment groups was similar to that found in adults, though no significance was found (p = 0.068). The mortality rates was 0% when oseltamivir administration was ≤ 2days, 1.0% when oseltamivir administration was 2–5days, 2.4% when oseltamivir administration >5 days after illness onset, and 6.4% in the control.

#### Cox regression analysis on the mortality risk of the different groups of antivirals

**Figure 2:**
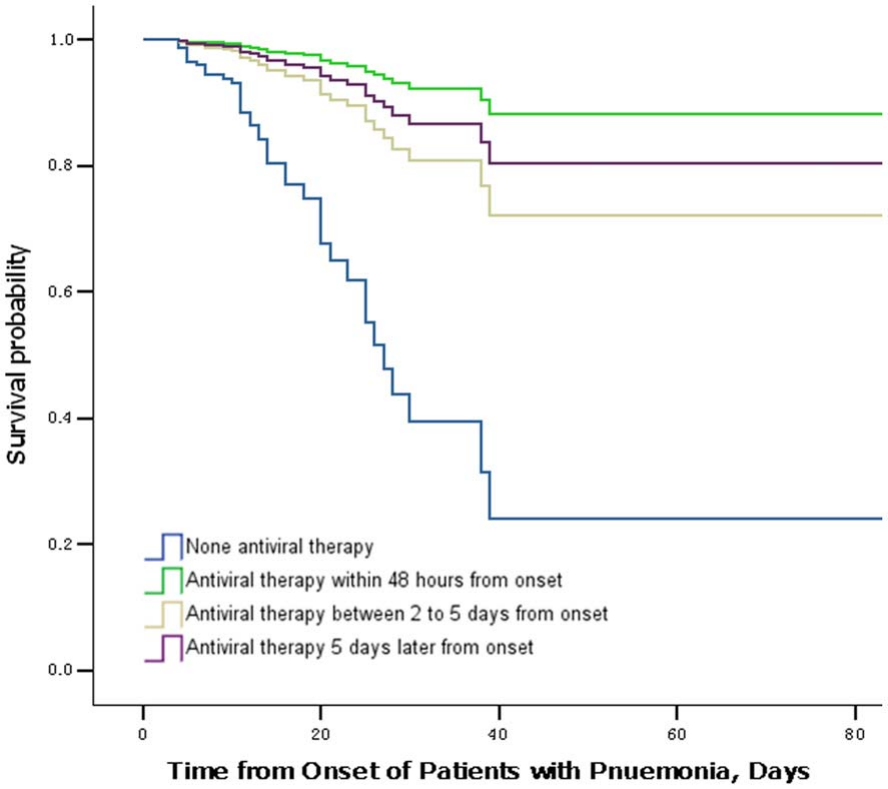
Cox regression for survival probability of pneumonia patients with antiviral therapy (Oseltamivir).

Cox regression analysis showed that: after 5 days from the onset of symptoms, the survival probability without antiviral therapy administration sharply decreased with a cumulative mortality of 6.3% (7 patients), while only 1, 1, and 0 patients died among patients who had antiviral therapy initiated within 48 hours, between 2–5 days, and >5 days after illness onset, respectively, All cumulative mortalities accounted for less than 0.5% of cases **(** **Figure 2** **)**. After 60 days after the onset of symptoms, 11 (10.8%) patients lacking antiviral therapy died, compared with 4 (1.8%) when oseltamivir was administered ≤ 2days, 18 (3.3%) 2–5days, and 23 (3.7%) >5 days after illness onset. The median time from illness onset to death was 6 (IQR: 5–11) days among patients who did not receive antiviral therapy, which was significantly shorter than among patients those who received oseltamivir ≤ 2days after symptom onset, with a median time of 18 (IQR: 6.5–68.8) days.

In comparison to patients who did not receive antiviral therapy, the crude mortality risk was reduced by 83% among patients who received oseltamivir (P<0.01). **(Figure S1)**.

**Figure 3:**
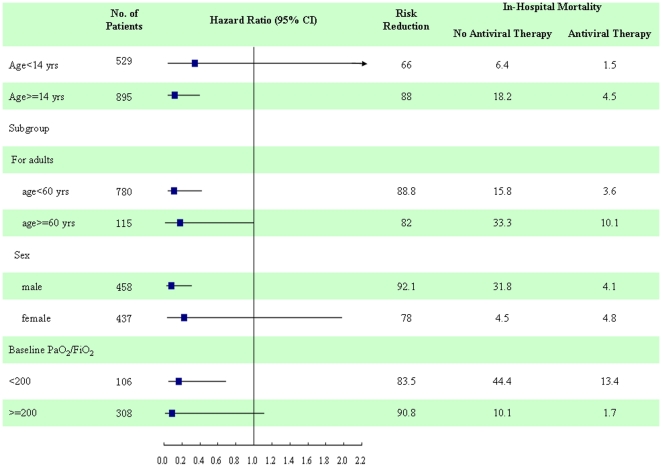
Estimates of hazard ratio for in-hospital mortality among patients with antiviral therapy, as compared with patients with no antiviral therapy. For patients aged<14 yrs or > = 14 yrs, adjusted for sex, and baseline APACHE II score; For age subgroup analysis among patients aged> = 14 yrs, adjusted for sex, and baseline APACHE II; for sex subgroup analysis among patients aged> = 14 yrs, adjusted for age, and baseline APACHE II; for baseline PaO2/FiO2, subgroup analysis among patients aged> = 14 yrs, adjusted for age, and sex.

For males, oseltamivir reduced the mortality risk by 92.1% (P<0.001). For patients aged ≥ 14 years, oseltamivir reduced the mortality risk by 88% (P<0.0001). Among adult patients, the protective effects of oseltamivir were greater in patients aged<60 years (P<0.05). For patients with baseline PaO2/FiO2<200, antiviral therapy with oseltamivir reduced the mortality risk by 83.5% (P<0.01). The risk reduction was 66% for children (age<14 years), 78% for female, and 90.8% for patients with baseline PaO2/FiO2≥200, but no significance was found **(** **Figure 3** **)**. Results of antiviral dosage and clinical outcomes are shown in **Table S4**. There was no difference of in-hospital mortality between patients treated with standard dose and higher dose.

## Discussion

This study has shown that the mortality due to pH1N1 viral pneumonia was 3.9%, a little lower than that of previous report [5]. The mortality due to pH1N1 viral pneumonia can be reduced by antiviral therapy (oseltamivir), even if treatment is initiated >48 hours after onset of illness. The findings have important therapeutic implications.

Oseltamivir is a potent and specific neuraminidase inhibitor for influenza viruses, and it inhibits replication of influenza A and B viruses in vitro [19]. However, studies of uncomplicated seasonal influenza cases have demonstrate that oseltamivir is effective only if administered within 48 h of the onset of symptoms [20]–[22]. Two recent observational studies have shown that treatment with oseltamivir may reduce mortality among hospitalized patients with pH1N1 influenza A, but both studies were limited by small sample sizes. One study in Mexico, which included 58 severe cases of pH1N1 influenza A infection and excluded patients who died within 72 hours of illness onset, found that survivors were more likely to have received treatment with a neuraminidase inhibitor than nonsurvivors (odds ratio 8.5, 95% CI 1.2–62.8) [16]. A second study from New York City has shown that the 28 hospitalized patients with pH1N1 influenza A who died were less likely to have received oseltamivir within two days of hospitalization than the 98 patients who survived (61 versus 96 percent) [23]. In this study, after excluding patients who died within 96 hours of illness onset, the in-hospital mortality was still higher in patients who did not have antiviral therapy compared with those who received oseltamivir treatment.

We agree that when managing patients suspected with influenza viral pneumonia, clinicians should not delay antiviral treatment while waiting for virological confirmation. In reality, some patients infected with influenza may begin to deteriorate 4 to 5 days after symptom onset [24]. In this cohort, median time from onset of illness to radiographic confirmation of pneumonia was 4 days; 83.4% of adults and 86% of children were initiated on antiviral therapy more than 48 hours from onset of illness. Our novel finding shows that oseltamivir treatment prescribed >2 days after onset still leads to survival benefit. As the viral load clearance may be delayed, severe patients may benefit from antiviral therapy even if initiated >48 hours after illness onset [25], [26]. Dr. Kelvin K. W et al demonstrated that in the ARDS-death group caused by influenza A pH1N1, nasopharyngeal influenza viral load decreased more slowly compared to mild diseases, even after oseltamivir therapy [27]. In our recent report, Influenza A pH1N1 virus RNA was still detectable in autopsy lung tissue from a 44-year-old previously healthy man 25 days after illness onset [28].

Greater protective effects of antiviral therapy among male patients, those aged 14 to 60 years, and patients with PaO2/FiO2<200 were our new findings. Several reasons should be considered. First, the Pneumonia Severity Index (PSI) scored the exact age for males and age minus 10 for females, meaning that males were more likely to die from pneumonia than females. Compared with females, males with severe pneumonia may benefit more from antiviral therapy [29]. Second, the Mexico study showed that 87% of death and 71% of cases of severe pneumonia involved patients between 5 and 59 years of age. While one third of the elderly subjects may have had cross-protective antibodies against the pH1N1 virus [30], this data may reflect an age shift to young and middle aged adults with severe disease as seen in previous pandemics. [31] Young adults with severe pneumonia may benefit more from antiviral therapy. Third, patients who died had worse hypoxemia [16], which may account for the greater protective effects of antiviral therapy among patients with ARDS.

In our study, we did not find more survival benefit when comparing administration of higher dose of oseltamivir compared to standard dose for patients with influenza A pH1N1 pneumonia. Our data suggested that for a patient weighing 40–78 kg, treatment with oseltamivir 150 mg Bid (higher dose) was not more effective than treatment with oseltamivir 75 mg Bid (standard dose), in terms of in-hospital mortality. However, for a patient who weighed more than 79 kg, oseltamvir 150 mg Bid was acceptable because such a dose was regarded as his/her “standard dose” (<3.8 mg/kg/d).

Our study has several limitations. Firstly, only a small number of cases (45 adults and 49 children) did not receive any antiviral therapy and acted as the “negative” control group. Secondly, despite the use of a prospective standardized data collection form, not all information was available for all patients.

In conclusion, this study of a large sample size has shown that antiviral therapy may improve survival in patients with severe pH1N1 viral pneumonia. Greater protective effects by antiviral therapy against fatality were found in male patients, patients aged 14 to 60 years, and patients with PaO2/FiO2<200. Administration of a double dose of oseltamivir for patients whose body weight was less than 78 kg conferred no additional survival benefit.

## Supporting Information

Figure S1 The estimates of hazard ratio for in-hospital mortality among patients with antiviral therapy, as compared with patients with no antiviral therapy. Adjusted for age, sex, baseline APACHE II score.
(TIF)

Figure S2 Classification criteria for of administration dose with oseltamivir. The definition of standard dose and higher dose was made based on frequency analysis of daily oseltamivir use. The dosage of antiviral therapy with oseltamivir was transposed according to each patient' body weight. Then the distribution of frequencies was described based on the patient's daily dosage per 1 kg of body weight. There were significant two peaks, which could be classified by 3.8 mg/kg/d (the mean daily dosage of oseltamivir).
(TIF)

Table S1 Demographic details and outcomes among pneumonia patients with complete oseltamivir treatment data (n = 1461).
(DOC)

Table S2 Antiviral therapy and outcomes of influenza pH1N1 viral pneumonia in adults. † Adult: age> = 14 ys. Data were presented as no./total no. (%), if otherwise stated. ‡ CNS system symptoms: refers to one or more of the following symptoms: insomnia, restlessness, hallucination, headache, dizziness and abnormal behaviour. §Acute renal failure: Serum Creatinine increased by 2-fold or GFR decreased >50%, or urine<0.5 ml/kg/h for at least 12 hours. ¶ Acute liver damage: AST or ALT >70 U/L, or Tbil >2 mg/dL. ※ Drug associated neuropsychological symptoms: refers to any neuropsychological symptoms which occurred during oseltamivir therapy in hospitals, such as insomnia, restlessness, hallucination, headache, dizziness and abnormal behaviour. ∮: Missing number was 1 among patients who were not prescribed active antiviral therapy, 7 among patients who were prescribed oseltamivir within 48 hours, 5 among patients who were prescribed oseltamivir 2–5days from onset, and 12 among patients who were prescribed oseltamivir 5 days later from onset. *P <0.05 and ** P<0.01. Comparison of antiviral therapy groups (Patients who received oseltamivir ≤ 2days, between 2–5 days and >5 days after illness onset) with control group (Patients who were not prescribed active anti-influenza therapy), by using Dunnett t (2-sided) test.
(DOC)

Table S3 Antiviral therapy and outcomes of influenza pH1N1 viral pneumonia in children £, China. £ Children: age<14 ys. Data were presented as no./total no. (%), if otherwise stated. † Immunosuppressant: patients with HIV/AIDS, or patients who were prescribed immunosuppressant agents, or corticosteroids (equivalent to prednisone 15 mg/d, 30 days). ‡ CNS system symptoms: refers to one or more of the following symptoms: insomnia, restlessness, hallucination, headache, dizziness and abnormal behaviour. §Acute renal failure: Serum Creatinine increased by 2-fold or GFR decreased >50%, or urine<0.5 ml/kg/h for at least 12 hours. ¶ Acute liver damage: AST or ALT >70 U/L, or Tbil >2 mg/dL. ※ Drug associated neuropsychological symptoms: refers to any neuropsychological symptoms which occurred during oseltamivir therapy in hospitals, such as insomnia, restlessness, hallucination, headache, dizziness and abnormal behaviour. *P<0.05 and ** P<0.01. Comparison of antiviral therapy groups (Patients who received oseltamivir ≤ 2days, between 2–5 days and >5 days after illness onset) with control group (Patients who were not prescribed active anti-influenza therapy), by using Dunnett t (2-sided) test.
(DOC)

Table S4 Antiviral dosage and outcomes of influenza pH1N1 viral pneumonia. † Standard dose, oseltamivir ≤ 3.8 mg/kg/d; higher dose, oseltamivir >3.8 mg/kg/d. ‡ Neuropsychological symptoms: refers to one or more of the following symptoms: insomnia, restlessness, hallucination, headache, dizziness and abnormal behaviour. §Acute renal failure: Serum Creatinine increased by 2-fold or GFR decreased >50%, or urine<0.5 ml/kg/h for at least 12 hours. ¶ Acute liver damage: AST or ALT >70 U/L, or Tbil >2 mg/dL. ※ Drug associated neuropsychological symptoms: refers to any neuropsychological symptoms which occurred during oseltamivir therapy in hospitals, such as insomnia, restlessness, hallucination, headache, dizziness and abnormal behaviour. *P<0.05 and ** P<0.01. Pairwise comparisons were performed using Dunnett t (2-sided) test. ∮: p = 0.830 for groups of lower dose and higher dose of oseltamivir. Missing number was 7 among patients who were not prescribed any active antiviral therapy, 15 among patients who were prescribed oseltamivir with lower dose, 10 among patients who were prescribed oseltamivir with higher dose.
(DOC)
