# Supplementary figures and images for: Antiviral Therapy and Outcomes of Patients with Pneumonia Caused by Influenza A Pandemic (H1N1) Virus
Source: PLoS One. 2012 Jan 20;7(1):e29652. doi: 10.1371/journal.pone.0029652 (PMC3262784; doi:10.1371/journal.pone.0029652)

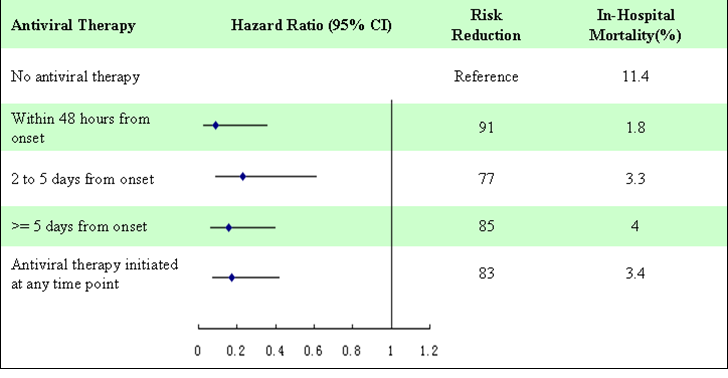

Supplement: Figure S1 — The estimates of hazard ratio for in-hospital mortality among patients with antiviral therapy, as compared with patients with no antiviral therapy. Adjusted for age, sex, baseline APACHE II score. (TIF) [file pone.0029652.s001.tif]

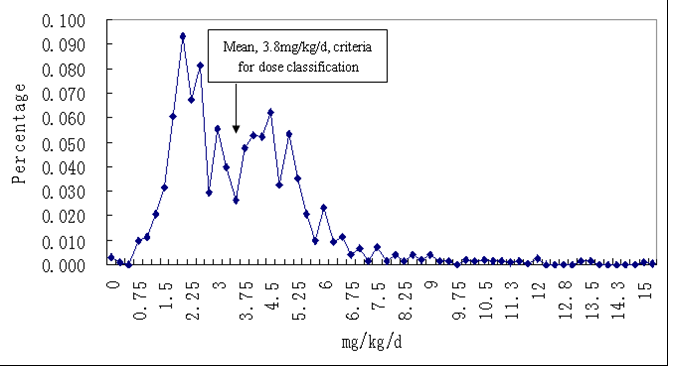

Supplement: Figure S2 — Classification criteria for of administration dose with oseltamivir. The definition of standard dose and higher dose was made based on frequency analysis of daily oseltamivir use. The dosage of antiviral therapy with oseltamivir was transposed according to each patient' body weight. Then the distribution of frequencies was described based on the patient's daily dosage per 1 kg of body weight. There were significant two peaks, which could be classified by 3.8 mg/kg/d (the mean daily dosage of oseltamivir). (TIF) [file pone.0029652.s002.tif]
