# Supplementary material for: Antiviral Therapy and Outcomes of Patients with Pneumonia Caused by Influenza A Pandemic (H1N1) Virus
Source: PLoS One. 2012 Jan 20;7(1):e29652. doi: 10.1371/journal.pone.0029652 (PMC3262784; doi:10.1371/journal.pone.0029652)
Supplement: Table S1 — Demographic details and outcomes among pneumonia patients with complete oseltamivir treatment data (n = 1461). (DOC) [file pone.0029652.s003.doc]

**Table S1**

Demographic details and outcomes among pneumonia patients with complete oseltamivir treatment data (n=1461)

| Variables | Findings |
| --- | --- |
| Gender-male | 807 (55.2) |
| Age (median, IQR, years) | 23.3 (5.2-41.3) |
| <14(%) | 541 (37.0) |
| 14-(%) | 803 (55.0) |
| >=60(%) | 117 (8.0) |
| Han Chinese (%) | 1,479 (95.5) |
| BMI > 30 (%) | 49(8.0) |
| Co-morbidities |  |
| Cardiovascular diseases (%) | 207 (13.4) |
| Respiratory diseases (%) | 164 (10.6) |
| Chronic renal diseases (%) | 34 (2.2) |
| Cirrhosis (%) | 15 (1.0) |
| Diabetes mellitus (%) | 91 (5.9) |
| Cancer and hematological diseases | 61 (4.0) |
| Stroke and Neuromuscular diseases | 25 (1.6) |
| Immunosuppressant † | 20 (1.3) |
| Pregnancy (%) | 119 (7.8) |
| Postmortum within 30 days after delivery | 44 (2.9) |
| Current smoker (%) | 180 (11.8) |
| Received seasonal or influenza A (H1N1) vaccination | 14 (1.0) |
| SARS history (%) | 3 (0.2) |
| Symptoms and Lab findings |  |
| T>=38 ℃ | 1271 (89.5) |
| cough | 1409 (96.4) |
| dyspnea | 407 (27.9) |
| hemoptysis | 90 (6.2) |
| CNS symptom | 129 (8.9) |
| WBC (×10^9^/L) | 6.8±3.8 |
| Platelet (×10^9^/L) | 194.3±80.1 |
| ALT >40U/L | 283 (21.1) |
| AST >40U/L | 527 (39.1) |
| LDH >300 u/L | 487 (39.6) |
| CK>200 u/L | 356 (28.5) |
| CRP (mg/L) | 50.0±104.0 |
| ESR (mm/h) | 32.2±26.0 |
| Antibiotics use | 1426 (97.6) |
| Antiviral plasma or convalescent plasma | 32 (2.2) |
| Mechanical ventilation | 143 (9.8) |
| Oseltamivir | 1367 (93.6) |
| Traditional Chinese Medicine | 750 (51.5) |
| Critical cases according to China influenza guideline | 288 (19.8) |
| Time from onset to discharge among survivors (median, IQR) | 14 (10-19) |
| Length of stay, median days, IQR | 9 (6-13) |
| Total cost, median dollars, IQR | 2319.5 (1264.2-4271.9) |
| Total In-hospital mortality (%) | 56(3.9) |
| For male (%) | 31(3.9) |
| For female (%) | 25(3.9) |
| For age <14 years (%) | 10(1.9) |
| For age >=14 & <60 years (%) | 33(4.2) |
| For age >=60 years (%) | 13(11.3) |
| Time from onset to death (mean ± SD, days) | 15.4 ±13.1 |
