# Supplementary material for: Antiviral Therapy and Outcomes of Patients with Pneumonia Caused by Influenza A Pandemic (H1N1) Virus
Source: PLoS One. 2012 Jan 20;7(1):e29652. doi: 10.1371/journal.pone.0029652 (PMC3262784; doi:10.1371/journal.pone.0029652)
Supplement: Table S2 — Antiviral therapy and outcomes of influenza pH1N1 viral pneumonia in adults. † Adult: age> = 14 ys. Data were presented as no./total no. (%), if otherwise stated. ‡ CNS system symptoms: refers to one or more of the following symptoms: insomnia, restlessness, hallucination, headache, dizziness and abnormal behaviour. §Acute renal failure: Serum Creatinine increased by 2-fold or GFR decreased >50%, or urine<0.5 ml/kg/h for at least 12 hours. ¶ Acute liver damage: AST or ALT >70 U/L, or Tbil >2 mg/dL. ※ Drug associated neuropsychological symptoms: refers to any neuropsychological symptoms which occurred during oseltamivir therapy in hospitals, such as insomnia, restlessness, hallucination, headache, dizziness and abnormal behaviour. ∮: Missing number was 1 among patients who were not prescribed active antiviral therapy, 7 among patients who were prescribed oseltamivir within 48 hours, 5 among patients who were prescribed oseltamivir 2–5days from onset, and 12 among patients who were prescribed oseltamivir 5 days later from onset. *P <0.05 and ** P<0.01. Comparison of antiviral therapy groups (Patients who received oseltamivir ≤ 2days, between 2–5 days and >5 days after illness onset) with control group (Patients who were not prescribed active anti-influenza therapy), by using Dunnett t (2-sided) test. (DOC) [file pone.0029652.s004.doc]

**Table S2.** Antiviral therapy and outcomes of influenza pH1N1 viral pneumonia in adults†

|  | No active anti-influenza therapy (n=45) | Oseltamivir within 48 hours from onset (n=145) | Oseltamivir 2-5days from onset (n=329) | Oseltamivir 5 days later from onset (n=401) | P value |
| --- | --- | --- | --- | --- | --- |
| Age (median, IQR, years) | 38 (24-50.8) | 28.2 (22.9-54.5) | 31.8 (23.4-46.9) | 38.7 (27.1-54.1) | 0.008 |
| Male sex n (%) | 23 (51.1) | 72 (49.7) | 158 (48.0) | 219 (54.6) | 0.343 |
| BMI>30 (%) n (%) | 2 (9.5) | 10 (9.3) | 18 (7.9) | 19 (7.4) | 0.936 |
| Any Underlying chronic diseases n (%) | 13 (28.9) | 56 (38.6) | 106 (32.2) | 136 (33.9) | 0.505 |
| Pregnancy n (%) | 2 (4.4) | 26 (18.3)* | 42 (12.8) | 41 (10.4) | 0.032 |
| Current smoker n (%) | 9 (20) | 20 (13.8) | 46 (14.2) | 82 (20.7) | 0.073 |
| **Symptoms and Lab findings and complications on admission** | | | | | |
| Hemoptysis n (%) | 1 (2.2) | 6 (4.1) | 27 (8.2) | 38 (9.5) | 0.096 |
| Dyspnea n (%) | 16 (35.6) | 38 (26.2) | 96 (29.2) | 139 (34.7) | 0.178 |
| CNS system symptoms‡ n (%) | 7 (15.6) | 11 (7.6) | 28 (8.5) | 33 (8.3) | 0.471 |
| Leucopenia ( < 4×10^9^/L) n (%) | 8 (19.0) | 20 (14.7) | 90 (28.8) | 92 (24.1) | 0.392 |
| ARDS n (%) | 9 (20.0) | 13 (9.0)* | 40 (12.2) | 47 (11.7) | 0.373 |
| Septic shock n (%) | 3 (8.6) | 3 (3.6) | 8 (3.5) | 8 (2.9) | 0.523 |
| Acute renal failure§ n (%) | 0 (0.0) | 1 (1.2) | 2 (0.9) | 4 (1.5) | 0.769 |
| Acute liver damage¶ n (%) | 4 (11.8) | 7 (8.1) | 28 (12.1) | 42 (14.5) | 0.429 |
| Laboratory evidence of bacterial co-infection n (%) | 1 (2.2) | 14 (9.7) | 20 (6.1) | 40 (10.0) | 0.07 |
| APACHE II score 24 hours admission (median, IQR) | 3 (2-7.5) | 6 (2-9) | 4 (2-8) | 4 (2-9) | 0.719 |
| SOFA score 24 hours admission (median, IQR) | 2 (0-4.5) | 1 (0-4) | 2 (0-4) | 2 (0.8-4) | 0.349 |
| **Treatment on admission** |  |  |  |  |  |
| Antibiotics n (%) | 43 (95.6) | 140 (96.6) | 322 (97.9) | 392 (97.8) | 0.716 |
| Traditional Chinese medicine n (%) | 25 (55.6) | 70 (49.0) | 171 (52.0) | 214 (53.5) | 0.782 |
| Oxygen therapy n (%) | 20 (44.4) | 71 (49.0) | 141 (42.9) | 204 (50.9) | 0.174 |
| Antiviral plasma or convalescent plasma n (%) | 0 (0) | 1 (0.7) | 5 (1.5) | 3 (0.7) | 0.547 |
| **Outcomes** |  | | | | |
| Mechanical ventilation n (%) | 6 (13.3) | 10 (6.9) | 27 (8.2) | 63 (15.7) | 0.003 |
| ICU admission n (%) | 7 (16.3) | 44 (31.4) | 76 (23.4) | 101 (25.6) | 0.148 |
| Drug associated neuropsychological symptoms※n (%) | 0 (0) | 6 (4.1) | 5 (1.5) | 6 (1.5) | 0.138 |
| Survival time for died patients(mean ± SD, days) | 8.2±4.9 | 9.6±10.0 | 10.5±7.6 | 12.9±10.1** | 0.00003 |
| In Hospital mortality∮n (%) | 8 (18.2) | 4 (2.9)** | 15 (4.6)** | 18 (4.9)** | <0.001 |

† Adult: age>=14ys. Data were presented as no./total no. (%), if otherwise stated.

‡ CNS system symptoms: refers to one or more of the following symptoms: insomnia, restlessness, hallucination, headache, dizziness and abnormal behaviour.

§Acute renal failure: Serum Creatinine increased by 2-fold or GFR decreased >50%, or urine<0.5ml/kg/h for at least 12 hours.

¶ Acute liver damage: AST or ALT > 70 U/L，or Tbil >2mg/dL.

※ Drug associated neuropsychological symptoms: refers to any neuropsychological symptoms which occurred during oseltamivir therapy in hospitals, such as insomnia, restlessness, hallucination, headache, dizziness and abnormal behaviour.

∮: Missing number was 1 among patients who were not prescribed active antiviral therapy, 7 among patients who were prescribed oseltamivir within 48 hours, 5 among patients who were prescribed oseltamivir 2-5days from onset, and 12 among patients who were prescribed oseltamivir 5 days later from onset.

*P <0.05 and ** P <0.01. Comparison of antiviral therapy groups (Patients who received oseltamivir ≤ 2days, between 2-5 days and >5 days after illness onset) with control group (Patients who were not prescribed active anti-influenza therapy), by using Dunnett t (2-sided) test.
