# Supplementary material for: Antiviral Therapy and Outcomes of Patients with Pneumonia Caused by Influenza A Pandemic (H1N1) Virus
Source: PLoS One. 2012 Jan 20;7(1):e29652. doi: 10.1371/journal.pone.0029652 (PMC3262784; doi:10.1371/journal.pone.0029652)
Supplement: Table S3 — Antiviral therapy and outcomes of influenza pH1N1 viral pneumonia in children £, China. £ Children: age<14 ys. Data were presented as no./total no. (%), if otherwise stated. † Immunosuppressant: patients with HIV/AIDS, or patients who were prescribed immunosuppressant agents, or corticosteroids (equivalent to prednisone 15 mg/d, 30 days). ‡ CNS system symptoms: refers to one or more of the following symptoms: insomnia, restlessness, hallucination, headache, dizziness and abnormal behaviour. §Acute renal failure: Serum Creatinine increased by 2-fold or GFR decreased >50%, or urine<0.5 ml/kg/h for at least 12 hours. ¶ Acute liver damage: AST or ALT >70 U/L, or Tbil >2 mg/dL. ※ Drug associated neuropsychological symptoms: refers to any neuropsychological symptoms which occurred during oseltamivir therapy in hospitals, such as insomnia, restlessness, hallucination, headache, dizziness and abnormal behaviour. *P<0.05 and ** P<0.01. Comparison of antiviral therapy groups (Patients who received oseltamivir ≤ 2days, between 2–5 days and >5 days after illness onset) with control group (Patients who were not prescribed active anti-influenza therapy), by using Dunnett t (2-sided) test. (DOC) [file pone.0029652.s005.doc]

**Table S3** Antiviral therapy and outcomes of influenza pH1N1 viral pneumonia in children ₤, China

|  | Patients who were not prescribed active anti-influenza therapy (n=49) | Patients who were prescribed oseltamivir within 48 hours from onset (n=69) | Patients who were prescribed oseltamivir 2-5days from onset (n=209) | Patients who were prescribed oseltamivir 5 days later from onset (n=214) | P value |
| --- | --- | --- | --- | --- | --- |
| Age (median, IQR, years) | 4.3 (1.2-8.8) | 5.1 (2.1-6.9) | 4.1 (2.5-6.0) | 3.8 (2.3-5.2) | 0.1335 |
| Male sex n (%) | 34(69.4) | 44(63.8) | 131(62.7) | 126(58.9) | 0.536 |
| Weight, (mean ± SD, kg) | 24.9±16.0 | 28.7±17.3 | 29.3±19.8 | 28.6±21.2 | 0.618 |
| Any Underlying chronic diseases n (%) | 8 (16.3) | 12 (17.4) | 35 (16.7) | 46 (21.5) | 0.600 |
| **Symptoms and Lab findings and complications on admission** | | | | | |
| Hemoptysis n (%) | 1 (2.0) | 1 (1.4) | 7 (3.3) | 9 (4.2) | 0.628 |
| Dyspnea n (%) | 5(10.2) | 18(26.1)* | 49(23.4) | 46(21.5) | 0.131 |
| CNS system symptoms‡ n (%) | 2(4.2) | 6(8.7) | 19(9.1) | 23(10.8) | 0.487 |
| Leucopenia ( < 4×10^9^/L) n (%) | 8 (17.4) | 5 (7.6) | 39 (20.2) | 48 (23.9) | 0.102 |
| Septic shock n (%) | 0(0.0) | 1(2.7) | 6(5.0) | 6(3.9) | 0.411 |
| Acute renal failure§ n (%) | 0(0.0) | 0(0.0) | 2(1.7) | 3(2.0) | 0.519 |
| Acute liver damage¶ n (%) | 2(6.5) | 2(5.4) | 8(6.6) | 15(9.6) | 0.725 |
| Laboratory evidence of bacterial co-infection n (%) | 0 (0) | 6 (8.7) | 5 (2.4) | 13 (6.1) | 0.015 |
| **Treatment on admission** | | | | | |
| Antibiotics n (%) | 48 (98.0) | 68 (98.6) | 203 (98.1) | 208 (97.2) | 0.893 |
| Traditional Chinese medicine n (%) | 24 (49.0) | 37 (53.6) | 101 (48.3) | 108 (51.2) | 0.866 |
| Oxygen therapy n (%) | 1 (2.0) | 7 (10.1) | 11 (5.3) | 18 (8.4) | 0.165 |
| Antiviral plasma or convalescent plasma n (%) | 1 (2.0) | 3 (4.4) | 7 (3.4) | 12 (5.6) | 0.558 |
| **Outcomes** |  | | | | |
| Mechanical ventilation n (%) | 1(2.0) | 7(10.1) | 11(5.3) | 18(8.4) | 0.165 |
| ICU admission n (%) | 2(4.2) | 17(25.0) | 29(14.4) | 69(32.5)** | <0.0001 |
| Drug associated neuropsychological symptoms※n (%) | 0 (0) | 0 (0) | 1 (0.5) | 1 (0.5) | 0.905 |
| Survival time for died patients(mean ± SD, days) | 8.7±7.2 | 7.8±3.5 | 9.0±5.1 | 11.7±8.2* | 0.00001 |
| In Hospital mortality n (%) | 3(6.4) | 0(0) * | 2(1.0) * | 5(2.4) | 0.068 |

₤ Children: age<14ys. Data were presented as no./total no. (%), if otherwise stated.

† Immunosuppressant: patients with HIV/AIDS, or patients who were prescribed immunosuppressant agents, or corticosteroids (equivalent to prednisone 15mg/d, 30 days).

‡ CNS system symptoms: refers to one or more of the following symptoms: insomnia, restlessness, hallucination, headache, dizziness and abnormal behaviour.

§Acute renal failure: Serum Creatinine increased by 2-fold or GFR decreased >50%, or urine<0.5ml/kg/h for at least 12 hours.

¶ Acute liver damage: AST or ALT > 70 U/L，or Tbil >2mg/dL.

※ Drug associated neuropsychological symptoms: refers to any neuropsychological symptoms which occurred during oseltamivir therapy in hospitals, such as insomnia, restlessness, hallucination, headache, dizziness and abnormal behaviour.

*P <0.05 and ** P <0.01. Comparison of antiviral therapy groups (Patients who received oseltamivir ≤ 2days, between 2-5 days and >5 days after illness onset) with control group (Patients who were not prescribed active anti-influenza therapy), by using Dunnett t (2-sided) test.
