# Supplementary material for: Antiviral Therapy and Outcomes of Patients with Pneumonia Caused by Influenza A Pandemic (H1N1) Virus
Source: PLoS One. 2012 Jan 20;7(1):e29652. doi: 10.1371/journal.pone.0029652 (PMC3262784; doi:10.1371/journal.pone.0029652)
Supplement: Table S4 — Antiviral dosage and outcomes of influenza pH1N1 viral pneumonia. † Standard dose, oseltamivir ≤ 3.8 mg/kg/d; higher dose, oseltamivir >3.8 mg/kg/d. ‡ Neuropsychological symptoms: refers to one or more of the following symptoms: insomnia, restlessness, hallucination, headache, dizziness and abnormal behaviour. §Acute renal failure: Serum Creatinine increased by 2-fold or GFR decreased >50%, or urine<0.5 ml/kg/h for at least 12 hours. ¶ Acute liver damage: AST or ALT >70 U/L, or Tbil >2 mg/dL. ※ Drug associated neuropsychological symptoms: refers to any neuropsychological symptoms which occurred during oseltamivir therapy in hospitals, such as insomnia, restlessness, hallucination, headache, dizziness and abnormal behaviour. *P<0.05 and ** P<0.01. Pairwise comparisons were performed using Dunnett t (2-sided) test. ∮: p = 0.830 for groups of lower dose and higher dose of oseltamivir. Missing number was 7 among patients who were not prescribed any active antiviral therapy, 15 among patients who were prescribed oseltamivir with lower dose, 10 among patients who were prescribed oseltamivir with higher dose. (DOC) [file pone.0029652.s006.doc]

**Table S4.** Antiviral dosage and outcomes of influenza pH1N1 viral pneumonia

|  | No active anti-influenza therapy (n=112) | Oseltamivir with standard dose† (n=632) | Oseltamivir with higher dose† (n=405) | P value |
| --- | --- | --- | --- | --- |
| Age (median, IQR, years) | 13.3 (3.8-37.8) | 27.8 (17.3-46.5)** | 5.2 (3.1-24.6)** | <0.001 |
| Male sex | 71 (63.4) | 344 (54.4) | 237 (58.5) | 0.139 |
| BMI>30 | 3 (6.1) | 46 (9.2) * | 2 (0.7) ** | <0.001 |
| Any Underlying chronic diseases | 22 (19.6) | 201 (31.8) ** | 75 (18.5) | <0.001 |
| Pregnancy | 5 (4.5) | 61 (9.8) | 22 (5.6) | 0.018 |
| Current smoker | 11 (10.1) | 99 (15.8) * | 19 (4.7) * | <0.001 |
| **Symptoms and Lab findings on admission** | | | | |
| Hemoptysis | 3 (2.7) | 45 (7.1) | 18 (4.5) | 0.053 |
| Dyspnea | 26 (23.2) | 161 (25.5) | 122 (30.1) | 0.173 |
| Neuropsychological symptoms‡ | 10 (9.0) | 63 (10.0) | 28 (6.9) | 0.221 |
| Leucopenia ( < 4×10^9^/L) | 18 (17.3) | 14.8 (24.6) | 72 (19.3) | 0.068 |
| ARDS | 10 (37.0) | 57 (21.8) | 46 (30.9) | 0.052 |
| Septic shock | 3 (3.7) | 13 (3.2) | 10 (4.1) | 0.815 |
| Acute renal failure§ | 0 (0) | 3 (0.7) | 3 (1.3) | 0.399 |
| Acute liver damage¶ | 7 (8.4) | 55 (12.9) | 30 (12.0) | 0.488 |
| APACHE II score 24 hours admission  (median, IQR) | 3 (1-6) | 4 (1-8) | 6 (3-9) | 0.01 |
| SOFA score 24 hours admission (median, IQR) | 0 (0-3) | 1 (0-4) | 2 (0-4) | 0.576 |
| Laboratory evidence of co-infection | 3 (2.7) | 37 (5.9) | 31 (7.7) | 0.105 |
| Antibiotics | 108 (96.4) | 615 (97.3) | 399 (98.5) | 0.292 |
| Traditional Chinese medicine | 54 (48.6) | 308 (48.9) | 211 (52.2) | 0.551 |
| Oxygen therapy | 28 (25.0) | 272 (43.0) | 150 (27.0) | 0.001 |
| Antiviral plasma or convalescent plasma | 1 (0.9) | 8 (1.3) | 16 (4.0) * | 0.011 |
| **Outcomes** |  | | | |
| Mechanical ventilation | 8 (7.1) | 47 (7.4) | 45 (11.1) | 0.109 |
| ICU admission | 16 (14.7) | 110 (17.7) | 116 (29.3) ** | <0.001 |
| Drug associated neuropsychological symptoms※ | - | 43 (6.8) | 23 (5.7) | 0.469 |
| In Hospital mortality∮ | 12 (11.4) | 17 (2.8) * | 10 (2.5) * | 0.001 |

† Standard dose, oseltamivir ≤ 3.8mg/kg/d; higher dose, oseltamivir > 3.8mg/kg/d.

‡ Neuropsychological symptoms: refers to one or more of the following symptoms: insomnia, restlessness, hallucination, headache, dizziness and abnormal behaviour.

§Acute renal failure: Serum Creatinine increased by 2-fold or GFR decreased >50%, or urine<0.5ml/kg/h for at least 12 hours.

¶ Acute liver damage: AST or ALT > 70 U/L，or Tbil >2mg/dL.

※ Drug associated neuropsychological symptoms: refers to any neuropsychological symptoms which occurred during oseltamivir therapy in hospitals, such as insomnia, restlessness, hallucination, headache, dizziness and abnormal behaviour.

*P <0.05 and ** P <0.01. Pairwise comparisons were performed using Dunnett t (2-sided) test.

∮: p=0.830 for groups of lower dose and higher dose of oseltamivir. Missing number was 7 among patients who were not prescribed any active antiviral therapy, 15 among patients who were prescribed oseltamivir with lower dose, 10 among patients who were prescribed oseltamivir with higher dose.
